# Occurrence and sequence analysis of porcine deltacoronaviruses in southern China

**DOI:** 10.1186/s12985-016-0591-6

**Published:** 2016-08-05

**Authors:** Shao-Lun Zhai, Wen-Kang Wei, Xiao-Peng Li, Xiao-Hui Wen, Xia Zhou, He Zhang, Dian-Hong Lv, Feng Li, Dan Wang

**Affiliations:** 1 Animal Disease Diagnostic Center, Institute of Animal Health, Guangdong Academy of Agricultural Sciences, Guangdong Key Laboratory of Animal Disease Prevention, Guangdong Open Laboratory of Veterinary Public Health, Guangzhou, 510640 China; 2 Department of Biology and Microbiology, South Dakota State University, Brookings, SD 57007 USA; 3 Department of Veterinary and Biomedical Science, South Dakota State University, Brookings, SD 57007 USA

**Keywords:** Porcine deltacoronavirus, Occurrence, Spike gene, Nucleocapsid gene, Sequence analysis, Southern China

## Abstract

**Background:**

Following the initial isolation of porcine deltacoronavirus (PDCoV) from pigs with diarrheal disease in the United States in 2014, the virus has been detected on swine farms in some provinces of China. To date, little is known about the molecular epidemiology of PDCoV in southern China where major swine production is operated.

**Results:**

To investigate the prevalence of PDCoV in this region and compare its activity to other enteric disease of swine caused by porcine epidemic diarrhea virus (PEDV), transmissible gastroenteritis coronavirus (TGEV), and porcine rotavirus group C (Rota C), 390 fecal samples were collected from swine of various ages from 15 swine farms with reported diarrhea. Fecal samples were tested by reverse transcription-PCR (RT-PCR) that targeted PDCoV, PEDV, TGEV, and Rota C, respectively. PDCoV was detected exclusively from nursing piglets with an overall prevalence of approximate 1.28 % (5/390), not in suckling and fattening piglets. Interestingly, all of PDCoV-positive samples were from 2015 rather than 2012–2014. Despite a low detection rate, PDCoV emerged in each province/region of southern China. In addition, compared to TGEV (1.54 %, 5/390) or Rota C (1.28 %, 6/390), there were highly detection rates of PEDV (22.6 %, 88/390) in those samples. Notably, all five PDCoV-positive piglets were co-infected by PEDV. Furthermore, phylogenetic analysis of spike (S) and nucleocapsid (N) gene sequences of PDCoVs revealed that currently circulating PDCoVs in southern China were more closely related to other Chinese strains of PDCoVs than to those reported in United States, South Korea and Thailand.

**Conclusions:**

This study demonstrated that PDCoV was present in southern China despite the low prevalence, and supported an evolutionary theory of geographical clustering of PDCoVs.

**Electronic supplementary material:**

The online version of this article (doi:10.1186/s12985-016-0591-6) contains supplementary material, which is available to authorized users.

## Background

Before 2012, the subfamily *Coronavirinae* included three genera (*Alphacoronavirus*,* Betacoronavirus *and *Gammacoronavirus*). However, in 2012, an emerging genus, *Deltacoronavirus*, was found in many animal species including swine from Hong Kong [1]. At present, more than five different coronaviruses have been described in swine populations. Among them, porcine epidemic diarrhea virus (PEDV), transmissible gastroenteritis virus (TGEV), and porcine respiratory coronavirus (PRCV) belong to the genus *Alphacoronavirus*; meanwhile, porcine hemagglutinating encephalomyelitis virus (PHEV) and Porcine deltacoronavirus (PDCoV) are assigned to the genus *Betacoronavirus * and the genus *Deltacoronavirus*, respectively [1]. Numerous studies have shown that more than half of porcine coronaviruses (including PDCoV) were enteropathogenic and caused acute diarrhea and vomiting in pigs, which resulted in huge economic losses for the global swine industry [2–6].

Currently, PDCoV has been reported in Hong Kong, North America, Mexico, South Korea, Thailand and some provinces of China [1, 7–19]. Despite recent progress, little is known about the prevalence and epidemiology of PDCoV in southern China (including Guangdong province, Hainan province, and Guangxi autonomous region), where major swine production is operated. Therefore, the aim of this study was to investigate the prevalence and sequence properties of PDCoV in this region.

## Methods

### Sampling

A total of 390 fecal samples (Table 1) were collected from 15 commercial swine farms with reported diarrhea in southern China. Farms A, D, E, J-O were from Guangdong province, Farms B, F, G were from Hainan province, and farms C, H, I were the Guangxi autonomous region. Farms A-I derived 30 samples with the following arrangement: ten samples from suckling piglets (<3 weeks old), ten samples from nursing piglets (between 3 and 9 weeks old), and ten samples from fattening piglets (>9 weeks old). The samples of farms A-I were collected between July and August 2015 and stored at −80 °C until further use. However, the samples of farms J-O were archived samples from 2012 to 2014. Prior to viral RNA extraction, fecal samples were diluted one time using Phosphate Buffered Saline (PBS) (pH: 7.4). The supernatants were then collected by centrifugation at 5000 × g for 5 min. 200 μl of clarified supernatants was used to extract viral RNA following the manufacturer’s recommendations (Axygen Scientific Inc.). RNA samples were stored at −80 °C until further analysis.

**Table 1:** Sample information and RT-PCR detection results of four diarrhea-associated viruses in pigs of various ages from 15 swine farms in southern China

Farm	Pigs (Age)	Number (*n*)	PDCoV	PEDV	TGEV	Rota C
A	Suckling piglets (<3 weeks old)	10	0/10	2/10	0/10	0/10
	Nursery pigs (>3 weeks old, <9 weeks old)	10	2/10	2/10	0/10	0/10
	Fattening pig (>9 weeks old)	10	0/10	1/10	0/10	0/10
B	Suckling piglets (<3 weeks old)	10	0/10	3/10	0/10	0/10
	Nursery pigs (>3 weeks old, <9 weeks old)	10	1/10	2/10	0/10	0/10
	Fattening pig (>9 weeks old)	10	0/10	0/10	0/10	0/10
C	Suckling piglets (<3 weeks old)	10	0/10	3/10	0/10	0/10
	Nursery pigs (>3 weeks old, <9 weeks old)	10	1/10	3/10	0/10	0/10
	Fattening pig (>9 weeks old)	10	0/10	0/10	0/10	0/10
D	Suckling piglets (<3 weeks old)	10	0/10	2/10	0/10	0/10
	Nursery pigs (>3 weeks old, <9 weeks old)	10	0/10	1/10	0/10	2/10
	Fattening pig (>9 weeks old)	10	0/10	1/10	1/10	0/10
E	Suckling piglets (<3 weeks old)	10	0/10	1/10	0/10	0/10
	Nursery pigs (>3 weeks old, <9 weeks old)	10	1/10	2/10	0/10	0/10
	Fattening pig (>9 weeks old)	10	0/10	0/10	0/10	0/10
F	Suckling piglets (<3 weeks old)	10	0/10	3/10	0/10	0/10
	Nursery pigs (>3 weeks old, <9 weeks old)	10	0/10	4/10	0/10	0/10
	Fattening pig (>9 weeks old)	10	0/10	1/10	2/10	0/10
G	Suckling piglets (<3 weeks old)	10	0/10	5/10	0/10	0/10
	Nursery pigs (>3 weeks old, <9 weeks old)	10	0/10	3/10	0/10	0/10
	Fattening pig (>9 weeks old)	10	0/10	0/10	0/10	0/10
H	Suckling piglets (<3 weeks old)	10	0/10	7/10	0/10	0/10
	Nursery pigs (>3 weeks old, <9 weeks old)	10	0/10	2/10	0/10	0/10
	Fattening pig (>9 weeks old)	10	0/10	0/10	0/10	0/10
I	Suckling piglets (<3 weeks old)	10	0/10	4/10	0/10	0/10
	Nursery pigs (>3 weeks old, <9 weeks old)	10	0/10	2/10	0/10	0/10
	Fattening pig (>9 weeks old)	10	0/10	2/10	0/10	0/10
J	Suckling piglets (<3 weeks old)	10	0/10	4/10	0/10	0/10
	Nursery pigs (>3 weeks old, <9 weeks old)	10	0/10	3/10	0/10	0/10
K	Suckling piglets (<3 weeks old)	10	0/10	5/10	0/10	2/10
	Nursery pigs (>3 weeks old, <9 weeks old)	10	0/10	5/10	2/10	0/10
L	Suckling piglets (<3 weeks old)	10	0/10	2/10	0/10	0/10
	Nursery pigs (>3 weeks old, <9 weeks old)	10	0/10	0/10	0/10	0/10
M	Suckling piglets (<3 weeks old)	10	0/10	2/10	0/10	0/10
	Nursery pigs (>3 weeks old, <9 weeks old)	10	0/10	3/10	0/10	0/10
N	Suckling piglets (<3 weeks old)	10	0/10	2/10	0/10	1/10
	Nursery pigs (>3 weeks old, <9 weeks old)	10	0/10	1/10	0/10	0/10
O	Suckling piglets (<3 weeks old)	10	0/10	2/10	0/10	0/10
	Nursery pigs (>3 weeks old, <9 weeks old)	10	0/10	3/10	1/10	0/10
Total		390	5/390	88/390	6/390	5/390

### Reverse transcription polymerase chain reaction (RT-PCR) detection

To detect PDCoV genome in collected fecal samples, the previously reported RT-PCR primers (41 F: 5’-TTTCAGGTGCTCAAAGCTCA-3’ and 735R: 5’-GCGAAAAGCATTTCCTGAAC-3’) targeting the nucleocapsid (N) gene with reaction conditions (50 °C for 30 min and 95 °C for 15 min for the reverse transcription reaction, followed by 40 cycles of PCR amplification at 95 °C for 15 s, 55 °C for 45 s, and 72 °C for 1 min, with a final extension at 72 °C for 7 min) were used [15]. In addition, molecular detection of the three diarrhea-related enteric viruses (Porcine epidemic diarrhea virus, PEDV; Porcine transmissible gastroenteritis virus, TGEV; Porcine rotavirus group C, Rota C) was performed in accordance with previous methods [20–22] for further evaluation of the possible co-infection status with PDCoV in investigated pig samples.

### Amplification of the spike (S) and N genes

To perform in-depth sequence comparison and phylogenetic analysis with known reference sequences (Additional file 1: Table S1), the complete spike (S) and N genes of PDCoV-positive samples were amplified according to previously published methods [8]. For amplification of the full-length S and N genes, previously reported RT-PCR primers (PDCoV-SF2: 5’-AGCGTTGACACCAACCTATT-3’ and PDCoV-SR2: 5’-TCGTCGACTACCATTCCTTAAAC-3’; PDCoV-NF1 : 5’-CCATC GCTCCAAG TCATTCT-3’ and PDCoV-NR1: 5’-TGGGTGGGTTTAACAGACATAG-3’) were used. PCR was carried out at 50 °C 30 min and 95 °C for 5 min, followed by 40 cycles of 98 °C for 10 s, 55 °C for 15 s, and 68 °C for 5 and 2 min for S and N genes, respectively; final extension was performed at 68 °C for 15 min. Positive amplicons were cloned into the pGM-19 T vector (Tiangen Inc. Beijing). Furthermore, all positive recombinant plasmids were submitted to a sequencing company (The Beijing Genomics Institute, BGI) and sequenced at least three times. Five S gene sequences and five N gene sequences were obtained (Additional file 1: Table S1), and have been submitted to GenBank database (accession numbers KU204694-KU204701, KX534090-KX534091).

### Phylogenetic analysis of the S and N genes

Sequence alignment analysis was performed using the Clustal W program implemented in DNAStar software. A phylogenetic tree was then constructed by the neighbor-joining method using the Molecular Evolutionary Genetics Analysis (MEGA) software version 5.1 with 1000 bootstrap replications set at 1000. Moreover, the possible recombination event was evaluated in the S and N genes by recombination detection program (RDP) 3.34 software.

## Results

### PDCoV detection

A total of 390 pig fecal samples, collected from 15 swine farms with reported diarrhea in southern China, were assessed for the presence of PDCoV and other viral enteric pathogens (PEDV, TGEV, and Rota C) by RT-PCR. As summarized in Table 1, the PDCoV genome was detected in specimens from 4 of 15 swine farms. Interestingly, the PDCoV genome was detected only in nursing piglets, and was absent in suckling piglets and fattening pigs. Although PDCoV was detected in each province/region of southern China, its overall prevalence in the investigated pigs of various age groups (*n* = 390) was relatively low (5/390, 1.28 %). The positive rate could be higher if only nursing piglets were included (5/150, 3.33 %). In contrast, the prevalence of PEDV, another porcine coronavirus causing epidemic diarrhea, was relatively higher (22.6 %, 88/390). In addition, PEDV was different from PDCoV in that it distributed similarly between nursing (36/150, 24 %) and suckling piglets (47/150, 31.3 %). Five fattening pigs from farms A, D, F and I were also tested positive for the PEDV genome. We also examined whether pigs with diarrhea harbored other enteric viruses such as TGEV and Rota C. Our results showed that the low detection rates (1.54 %, 5/390 for TGEV vs 1.28 %, 6/390 for Rota C) of the two pathogens were present in those pig samples. Intriguingly, co-infection of pigs by PDCoV and PEDV was observed (Table 1). All PDCoV positive nursing piglets were also tested positive for PEDV, thereby indicating a 100 % co-infection rate.

### Sequence comparison and phylogenetic analysis of the S gene of PDCoVs

**Fig. 1:**
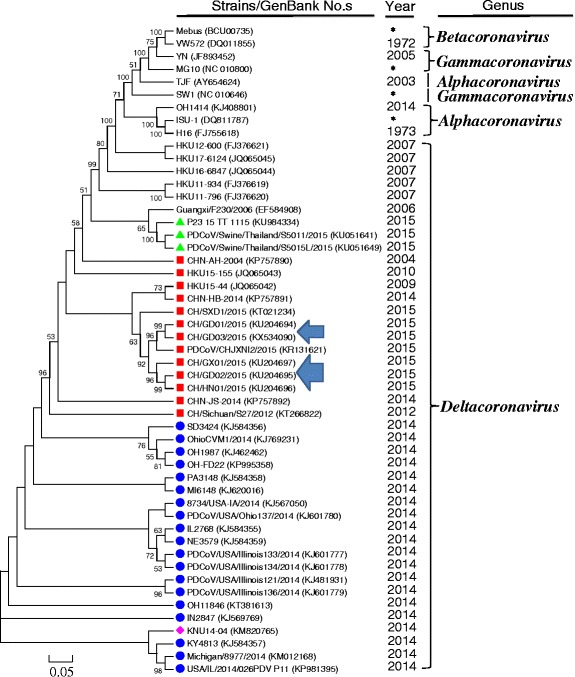
Phylogenetic analysis based on the S gene of PDCoVs and other coronaviruses. Note: Those PDCoV strains from China, America, South Korea and Thiland were labelled using “”, “”, “” and “”, respectively. Moreover, PDCoV strains in this study were labelled using left arrows. The collection time was not available for those coronavirus strains labelled using star symbols

The full-length sequences of S genes in five PDCoV-positive samples from the four different farms were amplified and designated provisionally CH/GD01/2015, CH/GD02/2015, CH/GD03/2015, CH/HN01/2015, and CH/GX01/2015, respectively. Sequencing results showed that they were composed of 3480 nucleotides (nt). Compared to all published American and individual Asian strains (including HKU15-44, CHN-AH-2004, KNU14-04, PDCoV/Swine/Thailand/S5015L/2015 and PDCoV/Swine/Thailand/S5011/2015), a 3-nt deletion in the S gene was identified in the five current PDCoV strains, six other Chinese viral strains and one Thai viral strain reported previously (Additional file 1: Table S1) [5, 17, 18]. Sequence alignment results revealed that CH/GD01/2015, CH/GD02/2015, CH/GD03/2015, CH/HN01/2015 and CH/GX01/2015 had >99.9 % homology in the S gene nucleotide sequence, indicating these five viral strains evolved from the same ancestor (Table 2). Further analysis showed that the five viruses reported in this study had the highest nucleotide identity (98.9 to 99.5 %) with a Jiangxi strain (PDCoV/CHJXNI2/2015) isolated from Jiangxi province bordering with the northern region of Guangdong province, and possessed the lowest nucleotide similarity (95.4 to 95.9 %) with PDCoV/Swine/Thailand/S5011/2015 and PDCoV/Swine/Thailand/S5015L/2015, isolated from Thailand (Table 2). From the above data, phylogenetic analysis of the S gene showed that the current PDCoVs circulating in southern China were most closely related to other Chinese PDCoV isolates than to those isolated previously from USA, South Korea and Thailand (Fig. 1). In addition, in the S gene, any possible recombinant events were not detected among those PDCoV strains.

**Table 2:** Nucleotide similarities (%) of S and N genes of our five PDCoV isolates and other reported PDCoVs and coronaviruses

		S gene					N gene				
GenBank No.		KU204694	KU204695	KU204696	KU204697	KX534090	KU204698	KU204699	KU204700	KU204701	KX534091
	Strain	CH/GD01/2015	CH/GD02/2015	CH/HN01/2015	CH/GX01/2015	CH/GD03/2015	CH/GD01/2015	CH/GD02/2015	CH/HN01/2015	CH/GX01/2015	CH/GD03/2015
KR131621	PDCoV/CHJXNI2/2015	99.5	98.9	98.9	99.1	99.2	99.7	99.1	99.1	99.3	99.4
KP757892	CHN-JS-2014	99.1	98.5	98.5	98.7	98.7	99.3	99.3	99.3	99.5	99.0
KP757891	CHN-HB-2014	98.9	98.4	98.4	98.7	98.5	98.8	98.6	98.6	98.8	98.5
JQ065042	HKU15-44	98.6	98.2	98.2	98.5	98.3	98.7	98.5	98.5	98.7	98.4
JQ065043	HKU15-155	98.5	98.2	98.2	98.4	98.2	98.7	98.5	98.5	98.7	98.4
KT021234	CH/SXD1/2015	98.4	97.9	97.9	98.1	98.0	99.0	98.8	98.8	99.0	98.7
KT266822	CH/Sichuan/S27/2012	98.4	98.0	98.0	98.2	98.0	99.2	99.0	99.0	99.2	98.9
KM820765	KNU14-04	98.4	97.9	97.9	98.2	98.1	98.8	98.6	98.6	98.8	98.5
KJ620016	MI6148	98.4	97.9	97.9	98.1	98.0	98.8	98.6	98.6	98.8	98.5
KJ584360	MN3092	-a	-a	-a	-a	-a	98.8	98.6	98.6	98.8	98.5
KJ584358	PA3148	98.4	98.0	98.0	98.2	98.0	98.8	98.6	98.6	98.8	98.5
KJ584357	KY4813	98.4	97.9	97.9	98.1	98.0	98.8	98.6	98.6	98.8	98.5
KJ584355	IL2768	98.4	97.9	97.9	98.1	98.0	98.8	98.6	98.6	98.8	98.5
KT381613	OH11846	98.4	97.9	97.9	98.1	98.0	98.8	98.6	98.6	98.8	98.5
KJ601779	PDCoV/USA/Illinois136/2014	98.4	97.9	97.9	98.1	98.0	98.5	98.3	98.3	98.5	98.3
KJ481931	PDCoV/USA/Illinois121/2014	98.4	97.9	97.9	98.1	98.0	98.5	98.3	98.3	98.5	98.3
KJ769231	OhioCVM1/2014	98.4	97.8	97.8	98.1	98.0	98.3	98.2	98.2	98.3	98.1
KJ601777	PDCoV/USA/Illinois133/2014	98.4	97.8	97.8	98.1	98.0	98.7	98.5	98.5	98.7	98.4
KJ584359	NE3579	98.4	97.8	97.8	98.1	98.0	98.7	98.5	98.5	98.7	98.4
KJ584356	SD3424	98.4	97.8	97.8	98.1	98.0	98.5	98.3	98.4	98.5	98.3
KJ462462	OH1987	98.4	97.8	97.8	98.1	98.0	98.7	98.5	98.5	98.7	98.4
KJ601778	PDCoV/USA/Illinois134/2014	98.3	97.8	97.8	98.0	98.0	98.7	98.5	98.5	98.7	98.4
KP995358	OH-FD22	98.3	97.8	97.8	98.0	98.0	-b	-b	-b	-b	-b
KJ601780	PDCoV/USA/Ohio137/2014	98.3	97.8	97.8	98.0	98.0	98.7	98.5	98.5	98.7	98.4
KJ569769	IN2847	98.3	97.8	97.8	98.1	98.0	98.8	98.6	98.6	98.8	98.5
KJ567050	8734/USA-IA/2014	98.3	97.9	97.9	98.1	98.0	98.7	98.5	98.5	98.7	98.4
KP981395	USA/IL/2014/026PDV_P11	98.3	97.8	97.8	98.0	97.9	98.7	98.5	98.5	98.7	98.4
KM012168	Michigan/8977/2014	98.3	97.8	97.8	98.0	97.9	98.7	98.5	98.5	98.7	98.4
KP757890	CHN-AH-2004	98.0	97.5	97.5	97.8	97.7	98.1	97.9	97.9	98.1	97.8
KU051641	PDCoV/Swine/Thailand/S5011/2015	95.8	95.6	95.6	95.9	95.4	97.1	96.9	96.9	97.1	96.8
KU051649	PDCoV/Swine/Thailand/S5015L/2015	95.8	95.5	95.5	95.9	95.4	97.1	96.9	96.9	97.1	96.8
KU984334	P23_15_TT_1115	95.9	95.8	95.6	95.9	95.5	97.8	97.8	97.8	98.0	97.5
EF584908	Guangxi/F230/2006	97.5	97.1	97.1	97.3	97.0	98.0	97.8	97.8	98.0	97.7
JQ065045	HKU17-6124	40.9	40.8	40.8	40.7	40.7	92.9	92.6	92.8	92.9	92.6
FJ376621	HKU12-600	40.7	41.1	41.1	41.0	40.6	76.4	75.7	76.2	76.3	76.3
JQ065044	HKU16-6847	57.0	56.9	56.9	57.0	57.2	75.1	75.3	75.2	75.4	75.1
FJ376619	HKU11-934	65.9	68.9	68.9	66.1	65.8	74.4	74.3	74.5	74.4	74.2
FJ376620	HKU11-796	65.3	65.6	65.7	65.6	65.1	74.4	74.6	74.5	74.4	74.2
KJ408801	OH1414 (PEDV)	38.2	38.3	38.3	38.1	37.8	3.6	3.5	3.5	3.6	3.6
FJ755618	H16(TGEV)	38.1	38.1	38.1	38.0	37.9	2.8	4.9	7.5	5.0	2.8
DQ811787	ISU-1(PRCV)	37.4	25.7	25.7	25.7	37.3	7.3	7.4	7.5	7.4	7.4
BCU00735	Mebus(bovine)	12.4	12.5	12.5	12.4	7.1	2.1	2.1	2.1	2.1	2.1
AY654624	TJF(SARS)	6.8	6.9	6.9	6.8	6.9	8.8	8.8	2.4	8.8	8.1
DQ011855	VW572(PHEV)	21.9	21.9	21.9	21.9	21.8	2.1	2.1	2.1	2.1	2.1
JF893452	YN(CIBV)	22.0	22.1	22.1	22.3	22.0	12.5	12.5	12.5	12.6	12.4
NC_010800	MG10 (Turkey)	24.1	23.5	23.5	23.3	24.0	14.0	14.0	14.0	14.1	14.1
NC_010646	SW1 (whale)	15.0	13.7	13.7	13.8	14.8	7.7	7.7	7.7	7.7	7.7

^a^S gene of MN3092 was not complete; ^b^S gene of OH-FD22 was not available

### Sequence comparison and phylogenetic analysis of the N gene of PDCoVs

**Fig. 2:**
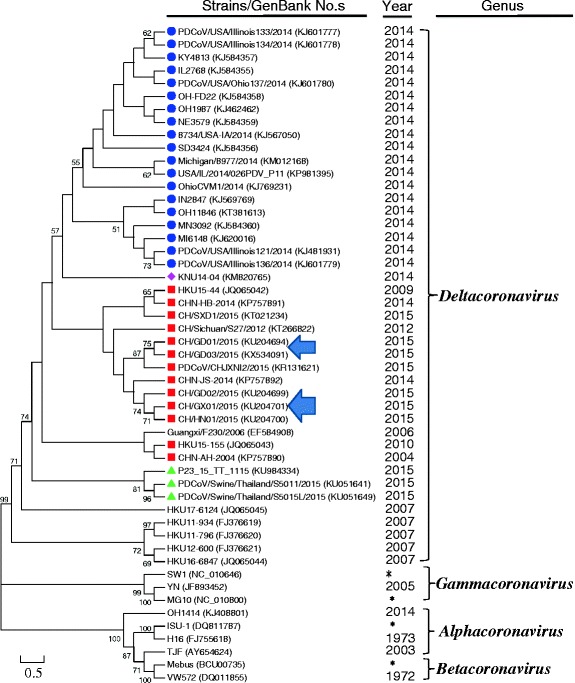
Phylogenetic analysis based on the N gene of PDCoVs and other coronaviruses. Note: Those PDCoV strains from China, America, South Korea and Thiland were labelled using “”, “”, “” and “”, respectively. Moreover, PDCoV strains in this study were labelled using left arrows. The collection time was not available for those coronavirus strains labelled using star symbols

Similarly, N gene sequences of all five PDCoV-positive samples were identified as 1029 nt in size. Sequence alignment results suggested that there was no deletion or insertion in N gene regions (Additional file 1: Table S1). Consistent with the data of S gene, multiple sequence alignment results of N gene showed that CH/GD01/2015, CH/GD02/2015, CH/GD03/2015, CH/HN01/2015 and CH/GX01/2015 had the highest nucleotide homology (99.1 to 99.7 %) with PDCoV/CHJXNI2/2015, and the lowest nucleotide homology (96.9 to 97.1 %) with PDCoV/Swine/Thailand/S5011/2015 and PDCoV/Swine/Thailand/ S5015L/2015 (Table 2). In addition, in the phylogenetic tree based on N gene, PDCoVs were divided into three main branches (Chinese branch, American branch and Thai branch). The five viral isolates reported from this work were clustered together within the Chinese branch (Fig. 2). Moreover, there were no any possible recombinant events occurring at the N gene of those PDCoV strains.

## Discussion

### Epidemiology of PDCoVs

PDCoV was first identified by *Deltacoronavirus* specific-PCR in rectal swabs of pigs (10.1 %, 17/169) with unknown healthy status in Hong Kong [1]. Then, PDCoV emerged in United States, China, South Korea and Thailand [5, 17, 18]. In most of studies, excluding PEDV, TGEV and porcine rotavirus, PDCoV, as an important enteric pathogen, was detected in clinical samples from pigs with diarrhea [9–12, 23]. In addition, it was confirmed experimentally that less than two-week old piglets were susceptible to PDCoV, which caused mild to moderate diarrhea as well as macroscopic and microscopic lesions in small intestines of conventional piglets (5-day-old), and severe diarrhea, vomiting, fecal shedding of virus, and severe atrophic enteritis in gnotobiotic pigs (11- to 14-day-old) [2, 3]. The data further confirmed that PDCoV were enteropathogenic in pigs. Meanwhile, PEDV or rotavirus showed higher detection rates in PDCoV-positive samples compared with other TGEV and rotavirus [8, 13–15, 24]. As shown above, co-infection of PDCoV and PEDV occurred in nursing piglets (Table 1), indicating that the diarrhea-related pathogens were quite complex clinically and not easy to control in the field. Moreover, in the two recent studies, PDCoV was shown to have higher infectivity in sows with diarrhea (81.0 %, 34/42) than nonclinical counterparts (23.5 %, 4/17) [8, 15], which might imply that sows often carry PDCoV. And further, it could result in the transmission of PDCoV from sows to the foetus and even newborn piglets, although the pathogenesis mechanism of PDCoV remains unclear.

To further understand the origin of PDCoV, some retrospective studies were made using PCR and enzyme-linked immunosorbent assay (ELISA) [24–26]. In Dong et al. [24] study, 2 of 6 samples collected from Anhui Province of China in 2004 were positive for PDCoV, up to now, it was the most ancient time for the detection of PDCoV in China. Meanwhile, PDCoV could date back to August 2013 in United States, where only 3 PDCoV-samples were detected using PCR in archived samples [25]. As for serology of PDCoV, anti-PDCoV IgG antibodies could date back to 2010 using an indirect anti-PDCoV IgG ELISA based on the putative S1 portion of the spike protein [26]. The above studies indicated that PDCoV could have circulated in China at least since 2004 and in United States since 2010. Maybe, due to limted samples in the present study, we did not detect PDCoV in pig samples collected from Guangdong province between 2012 and 2014. Although Asian leopard cat coronavirus (GenBank accession no. EF584908) was close to PDCoV in the phylogenetic trees (Figs. 1 and 2), in the future, more epidemiological surveys should be warranted to uncover the origin of PDCoV.

At the territory of China, Southern China mainly includes Guangdong province, Hainan province and the Guangxi autonomous region. Although molecular detection of PDCoV was performed in these regions [23, 24], little information was available on PDCoV prevalence. In a study by Chen et al. [23], an overall positive-PDCoV rate of 23.4 % (15/64) was obtained in all samples collected from Guangdong, Shanxi and Hubei provinces. However, more detailed data of PDCoV was not available in Guangdong province. Meanwhile, in the study from Dong et al. [24], only four archived samples from the Guangxi autonomous region were examined, but all negative for PDCoV. In this study, we demonstrated that PDCoV circulated and was co-infected by PEDV on those swine farms in Guangdong province, Hainan province and the Guangxi autonomous region, which further contributed to the epidemiology of PDCoV in these regions despite the relatively low prevalence.

### Genetic diversity of PDCoVs

The first two reported full-length PDCoV genome sequences (HKU15-44 and HKU15-155) were 25, 437 nt and 25, 432 nt in length, respectively [1], and they had 99.1 % nucleotide similarity with each other. Moreover, further sequence alignment showed a 3-nt (TAA) insertion in the S gene and a 3-nt (TTA) insertion in the 3’ untranslated region (UTR) of HKU15-44 [1, 5]. During the past 3 years, PDCoV-associated swine enteric disease was paid great attention in the major pig producing countries, especilly United States and China. Up to May 2016, more than 30 complete PDCoV genome sequences were published in the GenBank database. All were generated in China and United States except for one sequence from South Korea and three sequences from Thailand [8–19]. The Korean strain, KNU14-04, had 25, 422 nt in length, with similar genome features (a 3-nt insertion in the S gene with 3, 483 nt and a 3-nt insertion in the 3’ UTR, respectively) to all American strains and the Chinese strain (HKU15-44) [9]. Comparing complete genomes of the remaining Chinese strains to the American, Thai and Korean counterparts, CHN-HB-2014, CHN-JS-2014, PDCoV/CHJXNI2/2015, CH/Sichuan/S27/2012, CH/SXD1/2015 and P23_15_TT_1115 only had the 3-nt (AAT) deletion in the S gene (3, 480 nt) [14, 23, 24], while CHN-AH-2004 only had the 3-nt (TAA) deletion in the 3’ UTR [5, 24]. In the present study, 3-nt insertion was not found in UTR for our five obtained PDCoV (data not shown). Moreover, two additional unique features including a 6-nt (TTTGAA) deletion in the nonstructural protein (nsp) 2 region and a 9-nt (GCCGGTTGG) deletion in the nsp 3 region were also found in CH/Sichuan/S27/2012 [16]. However, for Thai viral isolates, they owned one additional unique nucleotide (C) insertion in the 3’ UTR [17, 18]. The biological significance of these naturally occurring deletions or insertions in PDCoV biology and pathogenesis warrants further investigations.

In this study, five S and five N gene sequences, respectively, were obtained to evaluate wherever genetic diversity of PDCoVs existed in southern China. Our results showed that these five S and five N gene sequences were more closely related to Chinese strains, and all clustered together in the phylogenetic tree (Table 2, Figs. 1 and 2). However, CH/GD01/2015 and CH/GD02/2015, reported in this study, originated from the same pig farm in Guangdong province, but had 48 nt and 12 nt differences in the S and N genes, respectively. The observed 48 nucleotide changes in the S gene made these viruses differ by 25 amino acid residues (Additional file 2: Table S2). For the N gene, the 12 nucleotide changes among these viruses resulted in 3 amino acid substitutions (Additional file 2: Table S2). Among them, 18 of 25 amino acid differences occurred at the first two-third parts of S gene. Interestingly, in spite of amino acid mutation, both S and N protein retained almost consistent amino acid properties (especially pH value) (Additional file 2: Table S2). Future study will address important roles of these polymorphisms in viral replication and pathogenesis. In addition, they were divided into two distinct small branches (Figs. 1 and 2). These findings suggested that PDCoVs in southern China have diverged from a common ancestor. Despite the emerging genetic diversity, overall, PDCoV prevalence is still largely restricted by the territory as demonstrated in Figs. 1 and 2.

For the two enteric coronaviruses (PEDV and TGEV) in pigs, the recombination events were often detected. However, most of them were from intra-recombination [27–30]. Recently, only one emerging recombinant/chimeric virus (named swine enteric coronavirus, SeCoV) was discovered in swine feces and resulted from inter-recombination of PEDV and TGEV, which had a TGEV backbone and a PEDV spike gene [31, 32]. In this study, there were no any possible recombinant events occurring in PDCoV strains. Maybe, the number and length of our obtained PDCoV sequences were limited. In the following study, the recombination event of PDCoV warrants further attentions.

## Conclusion

This study reported the prevalence of PDCoV on swine farms in southern China. Phylogenetic analysis of currently circulating PDCoV strains in this region and other previously reported strains supported the theory of geographical clustering of PDCoV infection landscape. The origin of various PDCoVs in different countries and regions should be further studied.

## Abbreviations

ELISA, enzyme-linked immunosorbent assay; MEGA, molecular evolutionary genetics analysis; N, nucleocapsid; PBS, phosphate buffered saline; PDCoV, porcine deltacoronavirus; PEDV, porcine epidemic diarrhea virus; PHEV, porcine hemagglutinating encephalomyelitis virus; PRCV, porcine respiratory coronavirus; RDP, recombination detection program; Rota C, porcine rotavirus group C; RT-PCR, reverse transcription polymerase chain reaction; S, Spike; SeCoV, swine enteric coronavirus; TGEV, transmissible gastroenteritis virus; UTR, untranslated region

## Additional files

Additional file 1: Table S1. Sequence information of PDCoVs and other coronaviruses used in the present study. (DOC 106 kb) Additional file 2: Table S2. AA differences of Spike (S) and Nucleocapsid (N) protein between CH/GD01/2015 and CH/GD02/2015. (DOC 42 kb)
