# Supplementary material for: Occurrence and sequence analysis of porcine deltacoronaviruses in southern China
Source: Virol J. 2016 Aug 5;13:136. doi: 10.1186/s12985-016-0591-6 (PMC4974758; doi:10.1186/s12985-016-0591-6)
Supplement: Additional file 1: Table S1. — Sequence information of PDCoVs and other coronaviruses used in the present study. (DOC 106 kb) [file 12985_2016_591_MOESM1_ESM.doc]

Additional file 1: Table S1 Information of PDCoVs in the present study and other reference coranaviruses

| GenBank Nos. | Strain | Virus | Host | Origin | Time | S gene (nt) | N gene (nt) |
| --- | --- | --- | --- | --- | --- | --- | --- |
| KU204694  KU204698 | CH/GD01/2015 | PDCoV | Pig | China: Guangdong | July-2015 | 3480 | 1029 |
| KU204695  KU204699 | CH/GD02/2015 | PDCoV | Pig | China: Guangdong | July-2015 | 3480 | 1029 |
| KU204696  KU204700 | CH/HN01/2015 | PDCoV | Pig | China: Guangdong | Augest-2015 | 3480 | 1029 |
| KU204697  KU204701 | CH/GX01/2015 | PDCoV | Pig | China: Guangdong | Augest-2015 | 3480 | 1029 |
| KX534090  KX534091 | CH/GD03/2015 | PDCoV | Pig | China: Guangdong | Augest-2015 | 3480 | 1029 |
| [KR131621](http://www.ncbi.nlm.nih.gov/nucleotide/919216900?report=genbank&log$=nuclalign&blast_rank=1&RID=53B3TKU401R) | PDCoV/CHJXNI2/2015 | PDCoV | Pig | China: Jiangxi | Mar-2015 | 3480 | 1029 |
| [KP757892](http://www.ncbi.nlm.nih.gov/nucleotide/807119229?report=genbank&log$=nuclalign&blast_rank=2&RID=53B3TKU401R) | [CHN-JS-2014](http://blast.ncbi.nlm.nih.gov/Blast.cgi#alnHdr_807119229) | PDCoV | Pig | China:Jiangsu | 20-Dec-2014 | 3480 | 1029 |
| [KP757891](http://www.ncbi.nlm.nih.gov/nucleotide/807119221?report=genbank&log$=nuclalign&blast_rank=3&RID=53B3TKU401R) | [CHN-HB-2014](http://blast.ncbi.nlm.nih.gov/Blast.cgi#alnHdr_807119221) | PDCoV | Pig | China: Hubei | 26-Dec-2014 | 3480 | 1029 |
| [JQ065042](http://www.ncbi.nlm.nih.gov/nucleotide/380005458?report=genbank&log$=nuclalign&blast_rank=4&RID=53B3TKU401R) | [HKU15-44](http://blast.ncbi.nlm.nih.gov/Blast.cgi#alnHdr_380005458) | PDCoV | Pig | China: Hong Kong | 2009 | 3483 | 1029 |
| [JQ065043](http://www.ncbi.nlm.nih.gov/nucleotide/380005466?report=genbank&log$=nuclalign&blast_rank=5&RID=53B3TKU401R) | [HKU15-155](http://blast.ncbi.nlm.nih.gov/Blast.cgi#alnHdr_380005466) | PDCoV | Pig | China: Hong Kong | 2010 | 3480 | 1029 |
| [KT021234](http://www.ncbi.nlm.nih.gov/nucleotide/927147686?report=genbank&log$=nuclalign&blast_rank=6&RID=53B3TKU401R) | [CH/SXD1/2015](http://blast.ncbi.nlm.nih.gov/Blast.cgi#alnHdr_927147686) | PDCoV | Pig | China: Shanxi | 20-Mar-2015 | 3480 | 1029 |
| [KT266822](http://www.ncbi.nlm.nih.gov/nucleotide/891151672?report=genbank&log$=nuclalign&blast_rank=7&RID=53B3TKU401R) | [CH/Sichuan/S27/2012](http://blast.ncbi.nlm.nih.gov/Blast.cgi#alnHdr_891151672) | PDCoV | Pig | China: Sichuan | 2012 | 3480 | 1029 |
| [KM820765](http://www.ncbi.nlm.nih.gov/nucleotide/695189892?report=genbank&log$=nuclalign&blast_rank=8&RID=53B3TKU401R) | [KNU14-04](http://blast.ncbi.nlm.nih.gov/Blast.cgi#alnHdr_695189892) | PDCoV | Pig | South Korea | Apr-2014 | 3483 | 1029 |
| [KJ620016](http://www.ncbi.nlm.nih.gov/nucleotide/643431439?report=genbank&log$=nuclalign&blast_rank=9&RID=53B3TKU401R) | [MI6148](http://blast.ncbi.nlm.nih.gov/Blast.cgi#alnHdr_643431439) | PDCoV | Pig | USA: Michigan | 18-Mar-2014 | 3483 | 1029 |
| KJ584360 | MN3092 | PDCoV | Pig | USA: Minnesota | 18-Feb-2014 | -b | 1029 |
| [KJ584358](http://www.ncbi.nlm.nih.gov/nucleotide/641452877?report=genbank&log$=nuclalign&blast_rank=10&RID=53B3TKU401R) | [PA3148](http://blast.ncbi.nlm.nih.gov/Blast.cgi#alnHdr_641452877) | PDCoV | Pig | USA: Pennsylvania | 18-Feb-2014 | 3483 | 1029 |
| [KJ584357](http://www.ncbi.nlm.nih.gov/nucleotide/641452869?report=genbank&log$=nuclalign&blast_rank=11&RID=53B3TKU401R) | [KY4813](http://blast.ncbi.nlm.nih.gov/Blast.cgi#alnHdr_641452869) | PDCoV | Pig | USA: Kentucky | 07-Mar-2014 | 3483 | 1029 |
| [KJ584355](http://www.ncbi.nlm.nih.gov/nucleotide/641452853?report=genbank&log$=nuclalign&blast_rank=12&RID=53B3TKU401R) | [IL2768](http://blast.ncbi.nlm.nih.gov/Blast.cgi#alnHdr_641452853) | PDCoV | Pig | USA: Ohio | 12-Feb-2014 | 3483 | 1029 |
| [KT381613](http://www.ncbi.nlm.nih.gov/nucleotide/938318404?report=genbank&log$=nuclalign&blast_rank=13&RID=53B3TKU401R) | [OH11846](http://blast.ncbi.nlm.nih.gov/Blast.cgi#alnHdr_938318404) | PDCoV | Pig | USA: Ohio | 07-May-2014 | 3483 | 1029 |
| [KJ601779](http://www.ncbi.nlm.nih.gov/nucleotide/645904420?report=genbank&log$=nuclalign&blast_rank=14&RID=53B3TKU401R) | [PDCoV/USA/Illinois136/2014](http://blast.ncbi.nlm.nih.gov/Blast.cgi#alnHdr_645904420) | PDCoV | Pig | USA: Illinois | 11-Jan-2014 | 3483 | 1029 |
| [KJ481931](http://www.ncbi.nlm.nih.gov/nucleotide/592930617?report=genbank&log$=nuclalign&blast_rank=15&RID=53B3TKU401R) | [PDCoV/USA/Illinois121/2014](http://blast.ncbi.nlm.nih.gov/Blast.cgi#alnHdr_592930617) | PDCoV | Pig | USA: Illinois | 04-Jan-2014 | 3483 | 1029 |
| [KJ769231](http://www.ncbi.nlm.nih.gov/nucleotide/668361756?report=genbank&log$=nuclalign&blast_rank=16&RID=53B3TKU401R) | [OhioCVM1/2014](http://blast.ncbi.nlm.nih.gov/Blast.cgi#alnHdr_668361756) | PDCoV | Pig | USA: Ohio | 01-Mar-2014 | 3483 | 1029 |
| [KJ601777](http://www.ncbi.nlm.nih.gov/nucleotide/645904404?report=genbank&log$=nuclalign&blast_rank=17&RID=53B3TKU401R) | [PDCoV/USA/Illinois133/2014](http://blast.ncbi.nlm.nih.gov/Blast.cgi#alnHdr_645904404) | PDCoV | Pig | USA: Illinois | 08-Jan-2014 | 3483 | 1029 |
| [KJ584359](http://www.ncbi.nlm.nih.gov/nucleotide/641452885?report=genbank&log$=nuclalign&blast_rank=18&RID=53B3TKU401R) | [NE3579](http://blast.ncbi.nlm.nih.gov/Blast.cgi#alnHdr_641452885) | PDCoV | Pig | USA: Nebraska | 21-Feb-2014 | 3483 | 1029 |
| [KJ584356](http://www.ncbi.nlm.nih.gov/nucleotide/641452861?report=genbank&log$=nuclalign&blast_rank=19&RID=53B3TKU401R) | [SD3424](http://blast.ncbi.nlm.nih.gov/Blast.cgi#alnHdr_641452861) | PDCoV | Pig | USA: South Dakota | 20-Feb-2014 | 3483 | 1029 |
| [KJ462462](http://www.ncbi.nlm.nih.gov/nucleotide/597710833?report=genbank&log$=nuclalign&blast_rank=20&RID=53B3TKU401R) | [OH1987](http://blast.ncbi.nlm.nih.gov/Blast.cgi#alnHdr_597710833) | PDCoV | Pig | USA: Ohio | 31-Jan-2014 | 3483 | 1029 |
| [KJ601778](http://www.ncbi.nlm.nih.gov/nucleotide/645904412?report=genbank&log$=nuclalign&blast_rank=21&RID=53B3TKU401R) | [PDCoV/USA/Illinois134/2014](http://blast.ncbi.nlm.nih.gov/Blast.cgi#alnHdr_645904412) | PDCoV | Pig | USA: Illinois | 08-Jan-2014 | 3483 | 1029 |
| [KP995358](http://www.ncbi.nlm.nih.gov/nucleotide/788262316?report=genbank&log$=nuclalign&blast_rank=22&RID=53B3TKU401R) | OH-FD22 | PDCoV | Pig | USA: Ohio | 20-Nov-2014 | 3483 | -a |
| [KJ601780](http://www.ncbi.nlm.nih.gov/nucleotide/645904428?report=genbank&log$=nuclalign&blast_rank=23&RID=53B3TKU401R) | [PDCoV/USA/Ohio137/2014](http://blast.ncbi.nlm.nih.gov/Blast.cgi#alnHdr_645904428) | PDCoV | Pig | USA: Ohio | 26-Jan-2014 | 3483 | 1029 |
| [KJ569769](http://www.ncbi.nlm.nih.gov/nucleotide/597710841?report=genbank&log$=nuclalign&blast_rank=24&RID=53B3TKU401R) | [IN2847](http://blast.ncbi.nlm.nih.gov/Blast.cgi#alnHdr_597710841) | PDCoV | Pig | USA: Ohio | 13-Feb-2014 | 3483 | 1029 |
| [KJ567050](http://www.ncbi.nlm.nih.gov/nucleotide/595644613?report=genbank&log$=nuclalign&blast_rank=25&RID=53B3TKU401R) | [8734/USA-IA/2014](http://blast.ncbi.nlm.nih.gov/Blast.cgi#alnHdr_595644613) | PDCoV | Pig | USA: Iowa | 20-Feb-2014 | 3483 | 1029 |
| [KP981395](http://www.ncbi.nlm.nih.gov/nucleotide/803360335?report=genbank&log$=nuclalign&blast_rank=28&RID=53B3TKU401R) | [USA/IL/2014/026PDV_P11](http://blast.ncbi.nlm.nih.gov/Blast.cgi#alnHdr_803360335) | PDCoV | Pig | USA: Illinois | 2014 | 3483 | 1029 |
| [KM012168](http://www.ncbi.nlm.nih.gov/nucleotide/686025218?report=genbank&log$=nuclalign&blast_rank=29&RID=53B3TKU401R) | [Michigan/8977/2014](http://blast.ncbi.nlm.nih.gov/Blast.cgi#alnHdr_686025218) | PDCoV | Pig | USA: Michigan | 17-Mar-2014 | 3483 | 1029 |
| [KP757890](http://www.ncbi.nlm.nih.gov/nucleotide/807119213?report=genbank&log$=nuclalign&blast_rank=32&RID=53B3TKU401R) | [CHN-AH-2004](http://blast.ncbi.nlm.nih.gov/Blast.cgi#alnHdr_807119213) | PDCoV | Pig | China: Anhui | 24-May-2004 | 3483 | 1029 |
| KU051641 | PDCoV/Swine/Thailand/S5011/2015 | PDCoV | Pig | Thailand | 10-Jun-2015 | 3483 | 1029 |
| KU051649 | PDCoV/Swine/Thailand/S5015L/2015 | PDCoV | Pig | Thailand | 30-Jun-2015 | 3483 | 1029 |
| KU984334 | P23_15_TT_1115 | PDCoV | Pig | Thailand | Nov-2015 | 3480 | 1029 |
| EF584908 | Guangxi/F230/2006 | Asian leopard cat coronavirus | Asian leopard cat | China: Guangxi | 2006 | 3108 | 1029 |
| JQ065045 | HKU17-6124 | Sparrow coronavirus | Sparrow | China: Hong Kong | 2007 | 3621 | 1029 |
| FJ376621 | HKU12-600 | Thrush coronavirus | Thrush | China: Hong Kong | Jan-2007 | 3579 | 1032 |
| JQ065044 | HKU16-6847 | White-eye coronavirus | White-eye | China: Hong Kong | 2007 | 3540 | 1044 |
| FJ376619 | HKU11-934 | Bulbul coronavirus | Bulbul | China: Hong Kong | Jan-2007 | 3498 | 1050 |
| FJ376620 | HKU11-796 | Bulbul coronavirus | Chinese bulbul | China: Hong Kong | Jan-2007 | 3492 | 1050 |
| KJ408801 | OH1414 | Porcine epidemic diarrhea virus | Pig | USA: Ohio | 23-Jan-2014 | 4161 | 1326 |
| FJ755618 | H16 | Transmissible gastroenteritis virus | Pig | China | 1973 | 4347 | 1149 |
| DQ811787 | ISU-1 | Porcine respiratory coronavirus | Pig | USA:Indiana | -a | 3669 | 1149 |
| BCU00735 | Mebus | Bovine coronavirus | Cattle | USA | -a | 4092 | 1347 |
| AY654624 | TJF | SARS coronavirus | Pig | China | 2003 | 3768 | 1269 |
| DQ011855 | VW572 | Hemagglutinating encephalomyelitis virus | Pig | Belgium | 1972 | 4050 | 1350 |
| JF893452 | YN | Infectious bronchitis virus | Chicken | China | 2005 | 3477 | 1230 |
| NC_010800 | MG10 | Turkey coronavirus | Turkey | Canada | -a | 3681 | 1230 |
| NC_010646 | SW1 | Beluga Whale coronavirus | Beluga Whale | USA | -a | 4419 | 1140 |

a not aviabile.

b S gene of MN3092 was not complete.
