# Supplementary material for: Occurrence and sequence analysis of porcine deltacoronaviruses in southern China
Source: Virol J. 2016 Aug 5;13:136. doi: 10.1186/s12985-016-0591-6 (PMC4974758; doi:10.1186/s12985-016-0591-6)
Supplement: Additional file 2: Table S2. — AA differences of Spike (S) and Nucleocapsid (N) protein between CH/GD01/2015 and CH/GD02/2015. (DOC 42 kb) [file 12985_2016_591_MOESM2_ESM.doc]

Additional file 2: Table S2 AA differences of Spike (S) and Nucleocapsid (N) protein between CH/GD01/2015 and CH/GD02/2015.

| Protein | Position | CH/GD01/2015 | CH/GD02/2015 |
| --- | --- | --- | --- |
| S | 43 | G | S |
|  | 51 | N | K |
|  | 60 | H | Y |
|  | 93 | F | L |
|  | 182 | I | T |
|  | 187 | V | L |
|  | 209 | G | D |
|  | 218 | T | K |
|  | 264 | V | L |
|  | 290 | A | D |
|  | 408 | V | I |
|  | 488 | Q | L |
|  | 505 | L | S |
|  | 540 | P | S |
|  | 622 | G | Q |
|  | 623 | A | V |
|  | 628 | S | P |
|  | 699 | P | S |
|  | 894 | Q | R |
|  | 938 | K | N |
|  | 1015 | I | V |
|  | 1061 | R | K |
|  | 1086 | L | W |
|  | 1111 | C | F |
|  | 1147 | D | N |
| N | 91 | A | S |
|  | 166 | I | T |
|  | 321 | L | M |

Note: Basic amino acids were labelled by red bands; Acidic amino acids were labelled by green bands; Neutral amino acids were not labelled.
